# Genomics of Invasive Cutibacterium acnes Isolates from Deep-Seated Infections

**DOI:** 10.1128/spectrum.04740-22

**Published:** 2023-03-28

**Authors:** Anna Both, Jiabin Huang, Moritz Hentschke, David Tobys, Martin Christner, Till Orla Klatte, Harald Seifert, Martin Aepfelbacher, Holger Rohde

**Affiliations:** a Institute for Medical Microbiology, Virology and Hygiene, University Medical Center Hamburg-Eppendorf, Hamburg, Germany; b Labor Doktor Fenner und Kollegen, Hamburg, Germany; c Institute for Medical Microbiology, Immunology and Hygiene, Faculty of Medicine and University Hospital Cologne, University of Cologne, Cologne, Germany; d German Center for Infection Research (DZIF), Partner Site Bonn-Cologne, Cologne, Germany; e Department for Trauma Surgery and Orthopedics, University Medical Center Hamburg-Eppendorf, Hamburg, Germany; University of Reims Champagne-Ardenne, Biomatériaux et Inflammation en site Osseux

**Keywords:** *Cutibacterium*, GWAS, foreign-material infection, genomics, invasion, opportunistic pathogens

## Abstract

Cutibacterium acnes, formerly known as Propionibacterium acnes, is a commensal of the human pilosebaceous unit but also causes deep-seated infection, especially in the context of orthopedic and neurosurgical foreign materials. Interestingly, little is known about the role of specific pathogenicity factors for infection establishment. Here, 86 infection-associated and 103 commensalism-associated isolates of *C. acnes* were collected from three independent microbiology laboratories. We sequenced the whole genomes of the isolates for genotyping and a genome-wide association study (GWAS). We found that *C. acnes* subsp. *acnes* IA_1_ was the most significant phylotype among the infection isolates (48.3% of all infection isolates; odds ratio [OR] = 1.98 for infection). Among the commensal isolates, *C. acnes* subsp. *acnes* IB was the most significant phylotype (40.8% of all commensal isolates; OR = 0.5 for infection). Interestingly, *C. acnes* subsp. *elongatum* (III) was rare overall and did not occur at all in infection. The open reading frame-based GWAS (ORF-GWAS) did not show any loci with a strong signal for infection association (no *P* values of ≤0.05 after adjustment for multiple testing; no logarithmic OR [logOR] of ≥|2|). We concluded that all subspecies and phylotypes of *C. acnes*, possibly with the exception of *C. acnes* subsp. *elongatum*, are able to cause deep-seated infection given favorable conditions, most importantly related to inserted foreign material. Genetic content appears to have a small effect on the likelihood of infection establishment, and functional studies are needed to understand the individual factors contributing to deep-seated infections caused by *C. acnes*.

**IMPORTANCE** Opportunistic infections emerging from human skin microbiota are of ever-increasing importance. Cutibacterium acnes, being abundant on the human skin, may cause deep-seated infections (e.g., device-associated infections). Differentiation between invasive (i.e., clinically significant) *C. acnes* isolates and sole contaminants is often difficult. Identification of genetic markers associated with invasiveness not only would strengthen our knowledge related to pathogenesis but also could open ways to selectively categorize invasive and contaminating isolates in the clinical microbiology lab. We show that in contrast to other opportunistic pathogens (e.g., Staphylococcus epidermidis), invasiveness is apparently a broadly distributed ability across almost all *C. acnes* subspecies and phylotypes. Thus, our work strongly supports an approach in which clinical significance is judged from clinical context rather than by detecting specific genetic traits.

## INTRODUCTION

Cutibacterium acnes, formerly known as Propionibacterium acnes, is a commensal of the human pilosebaceous unit. It is a non-motile, non-spore-forming rod-shaped Gram-positive anaerobic bacterium. Expression of cytochrome *d* oxidase genes facilitates tolerance to oxygen exposure for several hours and allows for growth even under conditions with limited oxygen (1). Sebum essentially supports *C. acnes* skin colonization, and thus, body sites rich in sebaceous glands (i.e., the face, shoulders, chest, axillae, and back) are more densely colonized with *C. acnes* compared to sites of the lower body (2). Males are colonized in greater numbers than females (3, 4). *C. acnes* has been implicated as a contributing factor in the inflammatory skin disease acne vulgaris, prostate cancer, postsurgical wound infection, and sarcoidosis (5).

Over the past decades, evidence demonstrates that *C. acnes* can also regularly cause infections associated with implanted medical devices (e.g., prosthetic joint infections [PJIs], prosthetic valve endocarditis [PVE], or cerebrospinal fluid [CSF] shunt infections), driven by the pathogen’s ability to form biofilms on artificial surfaces (6, 7). *C. acnes* may cause around 10% of prosthetic bone and joint infections, a number which some authors even consider underestimated (8). Typically, *C. acnes* infections clinically present with subacute courses, e.g., as joint implant loosening, prosthetic valve endocarditis, or cerebrospinal shunt infections (9–13). Despite its often gradual onset and low-grade nature, *C. acnes* infections can cause significant morbidity and loss of implant function, regularly warranting revision surgery (14).

Diagnosis of *C. acnes* infection, however, is challenging. Apart from its sometimes ambiguous clinical presentation, microbiological case definition is especially hampered by the ubiquitous presence of *C. acnes*, making the species also a common contaminant even of deep tissue specimens and despite appropriate skin disinfection measures (15–17). Moreover, recent research indicates that isolation of *C. acnes* even in high colony counts from prosthetic material of the shoulder does not necessarily correlate with symptomatic infection. Building on these findings, colonization of physiologically sterile sites by *C. acnes* without an inflammatory response has been suggested (18, 19).

As assessment of the pathogenic relevance of *C. acnes* isolated from normally sterile sites occurs at the nexus of contamination, colonization, and infection, the idea that commensal and disease-associated *C. acnes* isolates can be differentiated by defined markers has attracted significant interest. Biochemical and genetic studies recently allowed the division of *C. acnes* into three subspecies, *C. acnes* subsp. *acnes*, *C. acnes* subsp. *defendens*, and *C. acnes* subsp. *elongatum*, which were previously designated *C. acnes* types I, II, and III, respectively (20). *C. acnes* subsp. *acnes* is further divided into types IA_1_, IA_2_, IB, and IC. Previous work has shown that *C. acnes* subsp. *acnes* is particularly common in orthopedic implant infections, and type IB appears more important than the other types within *C. acnes* subsp. *acnes* (21). Interestingly, in acne vulgaris, *C. acnes* type IA appeared to more commonly elicit an inflammatory response (22, 23). Unfortunately, our knowledge of *C. acnes* pathophysiology is still quite limited. Genetic studies have helped to identify factors potentially inducing inflammation and chemotaxis in the genome (24, 25). Moreover, differences in the secretome of cultured *C. acnes* isolates belonging to different phylotypes have been shown (26). However, to date, no factors differentiating commensal or contaminating *C. acnes* isolates from infection isolates have been identified, and interpretation of isolates from clinical specimens remains difficult. This also relates to the fact that studies comparing the distribution of phylotypes in clinical specimens have had small sample sizes and usually do not take into account the abundance of respective phylotypes on the skin and thus their likelihood of gaining access to normally sterile body sites. Furthermore, functional genomic studies of *C. acnes* which could elucidate the contribution of specific genetic factors to pathogenesis are difficult and not widely applied in the research community (27).

Here, we ventured to prospectively collect proven invasive *C. acnes* isolates from three independent microbiology laboratories over the course of 3 years and to compare these isolates to contaminating isolates from blood cultures collected over the same period. A genome-wide association study (GWAS) was applied to search for factors associated with deep-seated infection.

## RESULTS

In total, 189 isolates recovered from individual patients were included. According to the study definitions (Table 1), 86 isolates were infection associated, of which 71 (82.6%) were isolated from bone and joint infections, 5 (5.7%) from soft tissue infections, 6 (6.9%) from endocarditis, and 4 (4.6%) from infections of the central nervous system (CNS) or eyes (Table 2). Seventy-three percent of infections were associated with foreign material, 8% were not associated with foreign material, and for 18.4%, the involvement of foreign-material was unknown. The control group comprised 103 commensal *C. acnes* isolates, which were isolated from a single blood culture. No patient data were collected for confidentiality reasons.

**TABLE 1:** Criteria for infection isolate collection^*a*^

Specimen source	Criteria
Native or prosthetic joint of the hip and knee	At least two independent positive specimens
	No incomplete wound healing or drains
	≥10 CFU/agar plate
	In case of sonication, >1,000 CFU/10 mL
Native or prosthetic shoulder joint	At least two independent positive specimens
	No incomplete wound healing or drains
	≥10 CFU/agar plate
	In case of sonication, >1,000 CFU/10 mL
	Histological signs of infection
Vertebral osteomyelitis	At least two independent positive specimens
	No incomplete wound healing or drains
	≥10 CFU/agar plate
Cerebrospinal fluid or brain tissue	Cytology and clinical signs suggestive of infection
	≥10 CFU/agar plate
Native or prosthetic heart valve	≥10 CFU/agar plate
Blood cultures	Clinical and echocardiographic signs of endocarditis
	≥3 positive blood cultures

a CFU, colony forming units.

**TABLE 2:** *C. acnes* isolate characteristics

Isolate type	No. (%) of infection isolates	No. (%) of contaminants
All infection sources	86	103
Bone and joint infections	71 (82.6)	NA^*a*^
Shoulder	27	
Vertebral	24	
Hip	7	
Knee	6	
Other	7	
Soft tissue infections	5 (5.7)	NA
Endocarditis	6 (6.9)	NA
CNS/ophthalmic infections	4 (4.6)	NA
*C. acnes* subsp. *acnes* (phylotype I)		
Type IA_1_	42 (48.3)	33 (32.4)
Type IA_2_	2 (2.3)	4 (3.9)
Type IB	22 (25.3)	42 (41.2)
*C. acnes* subsp. *defendens* (phylotype II)	19 (21.8)	15 (14.7)
*C. acnes* subsp. *elongatum* (phylotype III)	0 (0)	8 (7.8)
*C. acnes* subsp. nontypeable	2 (2.3)	1

a NA, not applicable.

### Genotyping results.

Aiming at elucidating the population structure of infection-associated and commensal isolates, phylotype subspecies and clonal complexes (CC) were determined based on draft genome sequences. Fisher’s exact test was used to assess the association of specific clades (phylotypes) and clonal complexes with either the infection or commensal isolation source.

**FIG 1:**
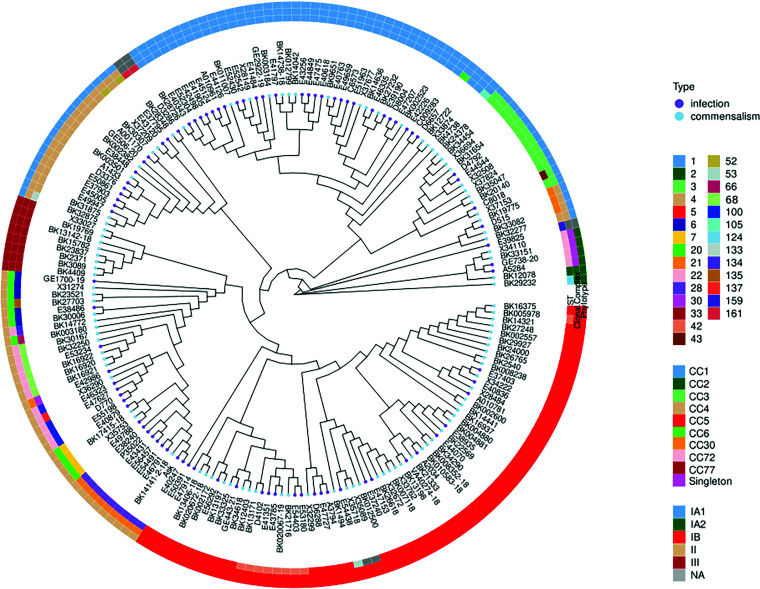
Phylogenetic tree of all isolates included in this study. For reasons of readability, the subspecies are not named but denoted as phylotypes. ST, sequence type; NA, not applicable.

**FIG 2:**
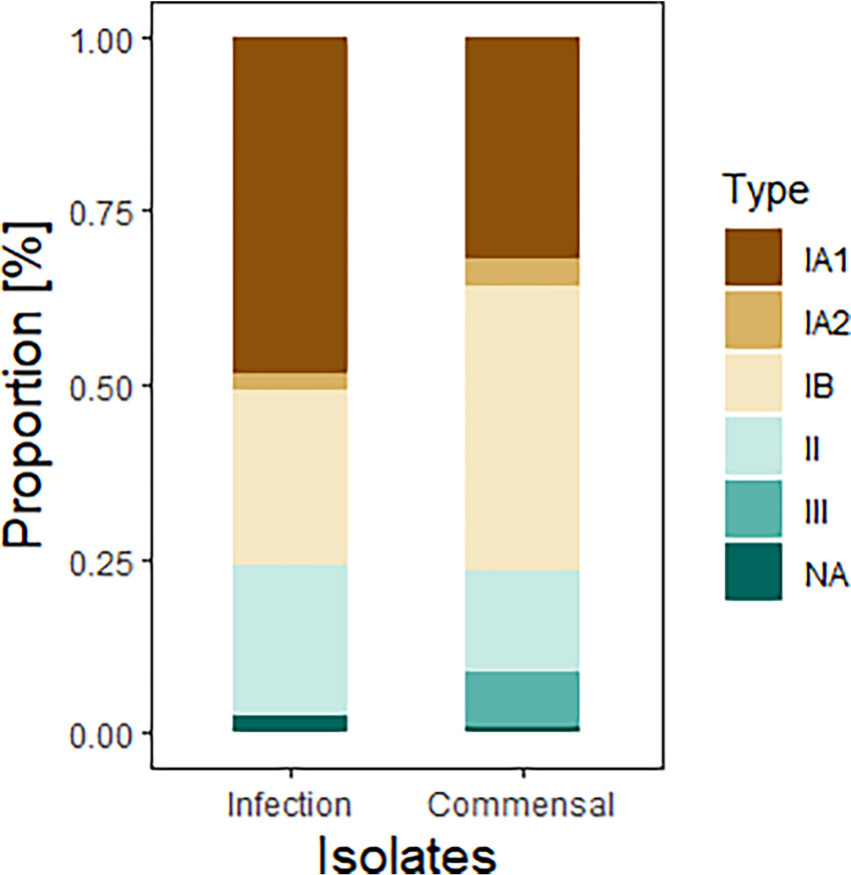
Distribution of phylotypes in infection-associated isolates and commensal isolates. Type II corresponds to *C. acnes* subsp. *defendens*, and type III corresponds to *C. acnes* subsp. *elongatum*.

Phylotyping was possible for 186 isolates; three isolates were at the edges of the tree and thus not typeable (Fig. 1). *C. acnes* subsp. *acnes* IA_1_ was the most common phylotype in infection isolates (48.3%) and significantly overrepresented in this group compared to the commensal isolates (odds ratio [OR], 1.98; 95% confidence interval [CI], 1.1 to 3.6; *P* = 0.025). On the other hand, *C. acnes* subsp. *acnes* (IB) and *C. acnes* subsp. *elongatum* (III) were significantly less common in the infection group; indeed, *C. acnes* subsp. *elongatum* (III) did not occur at all (OR, 0.5; 95% CI, 0.27 to 0.95; *P* = 0.03; and OR, 0.07; 95% CI, 0.08 to ≤ 0.001; *P* = 0.02, respectively) (Table 2; Fig. 2).

**FIG 3:**
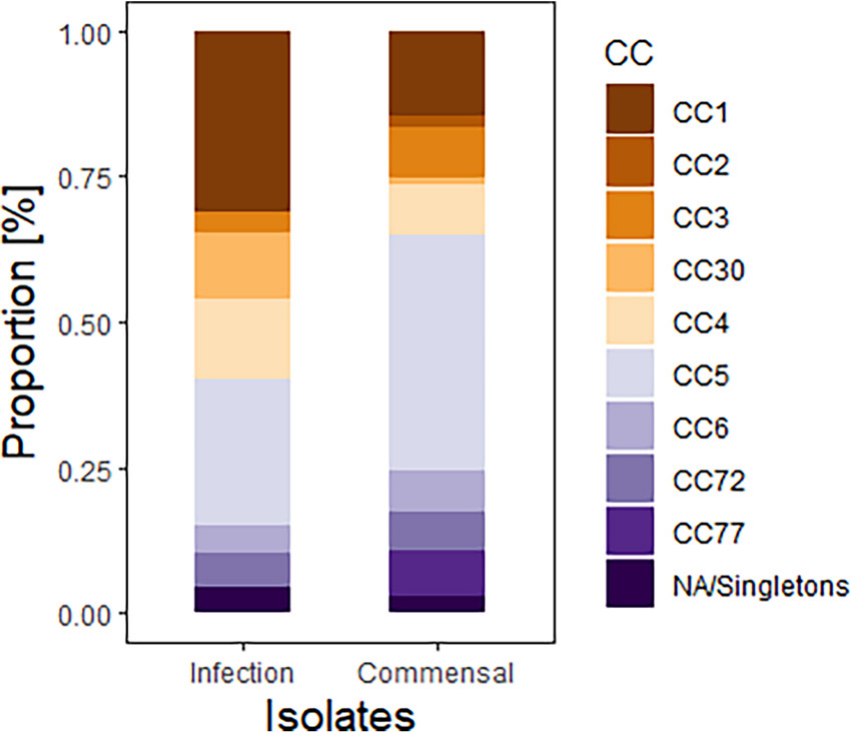
Distribution of clonal complexes in infection-associated isolates and commensal isolates.

The most common clonal complex in infection-associated isolates was CC1 (phylotype IA_1_), which along with CC30 (phylotype II) was significantly associated with infection (OR, 2.6; 95% CI, 1.3 to 5.4; *P* = 0.008; and OR, 13.2; 95% CI, 1.7 to 105.7; *P* = 0.003, respectively). CC5 (phylotype IB) and CC77 (phylotype III) were significantly more frequent in the commensal group (OR, 0.35; 95% CI, 0.26 to 0.92; *P* = 0.024; and OR, 0; 95% CI, 0 to infinity; *P* = 0.008, respectively) (Fig. 3).

Based on the finding that certain phylotypes and clonal complexes were more common in infection, we hypothesized that certain genomic traits facilitate infection establishment. In order to identify putative pathogenicity factors, an open reading frame (ORF)-based genome-wide association study (ORF-based GWAS) was conducted. ORFs were predicted from the pangenome of all *C. acnes* isolates sequenced for this study (infection associated, *n* = 86; commensal/blood culture contaminants, *n* = 103), and the presence or absence of each ORF was called for each isolate with a tolerance of 95% sequence similarity. In all, 565 ORFs had a native *P* value below 0.05, but after correction for multiple testing using the Benjamini-Hochberg false-discovery rate, no ORFs showed a significant *P* value (≤0.05), indicating that there was no ORF highly and confidently associated with infection. The number of tested ORFs in the GWAS was high, and thus the multiple testing burden was large. However, since many traits are linked, for example, through phylotype, the rather conservative correction may have led to an increase in false negatives (28). For that reason, we decided to examine ORFs with a native *P* value of ≤0.01. With this cutoff, 399 ORFs remained (91 infection associated and 308 commensalism associated). Of these, 299 could be explained exclusively by their presence (*n* = 256) or absence (*n* = 43) in *C. acnes* subsp. *elongatum* isolates, which we only found as commensal isolates, albeit at a very low frequency.

Assessing the remaining 100 ORFs, the maximum accuracy with which a single ORF could predict infection and commensalism was 0.62. No ORFs had a logarithmic odds ratio (logOR) of ≥2 or less than or equal to −2, which would indicate a high effect size (29). Six ORFs had a logOR of ≥1 (*n* = 2) or less than or equal to −1 (*n* = 4) for infection association, indicating a moderate effect size (Table 3; see also Data set S2 in the supplemental material).

**TABLE 3:** Infection- and commensalism-associated ORFs^*a*^

ORF	NCBI protein accession no. (reference genome)	Function	Native *P* value	logOR
Group_2480	None	Hypothetical protein	0.002	−1.13
Group_2170	None	Hypothetical protein	0.004	−1.09
Group_2694	None	Hypothetical protein	0.004	−1.09
Group_8787	None	Hypothetical protein	0.004	−1.09
*ortA*	AVT27627.1	Hypothetical protein	0.007	1.01
Group_1940	AVT26695.1	Polyhydroxyalkanoate synthase	0.004	1.09

a The sequences of the predicted ORFs can be found in Data set S2 in the supplemental material.

The alignment of all ORFs with a native *P* value of ≤0.01 to reference genomes of phylotypes IA_1_ (ATCC 6919; GenBank accession number CP023676.1), IB (PA15_2_L1; CP012351.1), and II (ATCC 11828; CP003084.1) showed that most of the significantly associated ORFs were evenly distributed over the whole genome. However, we found a cluster in a phylotype IB-specific genomic region with commensalism-associated ORFs (14 ORFs, nucleotides 2233281 to 2250990 in the reference genome found under GenBank accession number CP012351.1) (Fig. S1 to S3). The physiological importance of this cluster is unfortunately unclear, and the genomic region may be a characteristic of phylotype IB rather than a virulence mitigating trait.

The GWAS model which we used was built to take into account population structure by pairwise comparisons. However, most ORFs from the accessory genome were in linkage disequilibrium and would pass the significance threshold together; thus, phylogeny still had an important impact on the GWAS results, and interpretation of the ORF importance was mostly impossible without functional data and annotation. In order to reduce the impact of phylotype on the GWAS, we repeated the GWAS separately for phylotypes IA_1_ (infection-associated isolates, *n* = 42; commensalism-associated isolates, *n* = 33), IB (infection associated, *n* = 22; commensalism associated, *n* = 42), and II (infection associated, *n* = 18; commensalism associated, *n* = 15) (Table S3). There were no ORFs associated with infection in more than one phylotype and thus no indication of a phylotype-independent pathofactor.

## DISCUSSION

Cutibacterium acnes is an important cause of subacute foreign-material associated infections, both in orthopedic, cardiac, and neurosurgical contexts. Previously, different factors were implicated in facilitating the transition from commensalism in the pilosebaceous unit to the infection environment, including biofilm formation and specific genetic backgrounds (i.e., association with defined phylotypes and clonal complexes) (30, 31).

*In vitro* experiments have shown that *C. acnes* possesses several putative virulence factors, such as assembly of polysaccharide-based biofilms, proinflammatory mechanisms (e.g., production of free fatty acids through lipase expression, for example, GehA and GehB [32]), coproporphyrin III secretion (1), or expression of extracellular matrix recognizing adhesion factors and pore-forming toxins (CAMP1 to CAMP5) (1). Interestingly, both CAMP expression and lipase expression have shown phylotype-dependent variability (32, 33). However, the importance of these factors has mainly been studied in acne vulgaris, while their specific contribution to the pathogenesis of implant-associated *C. acnes* infections remains unclear. The role of specific *C. acnes* pathogenicity factors in symptomatic deep-seated infection is particularly intriguing as *C. acnes* can be recovered in meaningful amounts from physiologically sterile sites with or without signs of inflammation. For example, it has been shown that *C. acnes* is a major cause of symptomatic shoulder PJI; at the same time, *C. acnes* strains are also isolated with high frequency from osteosynthetic material of the clavicular bone without any signs of infection or inflammation in the patient (18, 34).

Previously, high-resolution genomic approaches comparing true invasive and colonizing bacterial populations have been instrumental in dissecting the genetic factors associated with or even functional for opportunistic pathogenicity in commensal organisms. Herein, we present the first study employing large-scale genomics to understand the opportunistic pathogenicity of *C. acnes* and to fill essential gaps in our understanding of the genetic requirements necessary for *C. acnes* to cause infection.

A similar distribution of phylotypes of commensal *C. acnes* isolates was found as in previous studies, with *C. acnes* subsp. *acnes* IA_1_ being the most abundant phylotype (35). Furthermore, *C. acnes* subsp. *acnes* (IB) and *C. acnes* subsp. *defendens* (II) were commonly encountered, while phylotypes *C. acnes* subsp. *acnes* (IA_2_) and *C. acnes* subsp. *elongatum* (III) were rare. Interestingly, the diversity of commensal phylotypes was largely maintained in infection isolates. With the exception of *C. acnes* subsp. *elongatum* (III), which did not occur in infection, all other phylotypes present in noninvasive contaminants were also identified in disease-associated isolates. Though phylotype IA_1_ was significantly associated with infection and phylotype IB was significantly more common in commensal strains, the odds ratios were not more than double in either one. Of note, *in vitro* studies have found IA_1_ to produce more biofilm than other phylotypes (36). This may at least partially explain the phylotype’s increased frequency in disease-causing isolates. In conflict with our findings, though, a smaller study from Sweden comparing phylotypes of *C. acnes* isolates from 63 patients suffering from PJI and from the skin of 56 healthy individuals did not find any significant differences between the two groups (37), while other studies noted a higher number of IB and II in foreign material-associated and orthopedic infection (21, 35). In one study, phylotype IA_1_ was more frequently detected in CNS infection (31). In summary, the overall effect of a given phylotype on the likelihood of invasive disease appears rather small. Possibly with the exception of *C. acnes* subsp. *elongatum*, all phylotypes seem to be able to cause infection in the presence of specific host risk factors and the opportunity to enter a sterile site conducive to biofilm formation. Of note, only 8% of infections observed in this study occurred out of the context of foreign materials, with no predilection for any phylotype, supporting the idea that host factors (i.e., implanted medical devices) are of essential importance for invasive *C. acnes* infections to occur. Small phylotype-specific differences in pathogenic potential may be present; however, larger observational clinical studies and more research into the pathophysiology of *C. acnes* in deep-seated infection are warranted to appreciate such subtle differences.

To unravel potential phylotype-independent genetic loci associated with *C. acnes* invasive potential, an ORF-based GWAS was conducted; however, it failed to detect any strong signals for infection or commensalism association (no significant loci after adjustment for multiple testing). We then lowered our threshold for significance, as the Benjamini-Hochberg procedure can be quite conservative and mute weaker effects. Discounting ORFs that were solely associated with *C. acnes* subsp. *elongatum*, 100 loci had a *P* value of ≤0.01. The effect sizes of individual loci were rather low (no loci with a logOR value of ≥|2| and *P* ≤ 0.01), compared to GWAS from other bacterial species, which analyzed gene content based on infection association (38, 39). Some of these factors may facilitate infection establishment to some degree. However, the pathophysiological importance of each one cannot be discerned from the GWAS results alone, as loci may be linked to possibly pathophysiologically important factors through linkage disequilibrium, rather than being pathofactors themselves. Furthermore, the likelihood of invasiveness may be multifactorial, and the combination of several loci, as well as differences in gene expression, may contribute to pathogenic potential. Obviously, more functional studies are needed to further clarify *C. acnes* infection establishment. Based on the evidence presented here, future studies should broaden their perspective to include proteomic and metabolic approaches, as accessory gene content alone does not appear decisive for infection establishment.

As a limitation of this study, we need to address the comparison group of contaminating *C. acnes* isolates. We selected isolates from contaminated blood cultures of hospital patients, without regard to their duration of stay. Thus, we cannot exclude that these isolates may in some way be different from isolates collected in the community. To our knowledge, no hospital-adapted strains of *C. acnes* have thus far been described, but this possibility cannot be excluded and should be further investigated in the future.

In conclusion, this study analyzed a large number of infection- and commensalism-associated isolates of *C. acnes* collected by three independent microbiology departments. We found evidence for phylotype-dependent differences in pathogenic potential; however, it was not possible to pin this effect on individual pathogenetic factors. Surgery of the trunk involving foreign material implantation appears to be the most important risk factor for development of *C. acnes* infections, regardless of the underlying phylotype.

## MATERIALS AND METHODS

### Study setting and isolate collection.

Two medical microbiology laboratories localized at German university medical centers and one major privately owned laboratory serving several hospitals with orthopedics departments participated in the study. The criteria used to define infection-associated isolates were as specified in Table 1. Additionally, isolates from single positive blood cultures without repeated isolation of *C. acnes* from the same patient and no clinical signs of localized infection were included as the control group (commensal isolates). Participating microbiologists at each laboratory independently decided whether inclusion criteria had been met and submitted isolates to the University Medical Centre Hamburg-Eppendorf for further analysis. The corresponding diagnosis was submitted with the isolates, as well as the specimen source site, number of collected specimens from the affected site, and number of *C. acnes* culture-positive specimens, but no patient data were submitted in order to ensure anonymous handling of the isolates by the researchers analyzing the data. Upon arrival, the isolates were cultured on Schaedler agar (Oxoid, Basingstoke, UK) for 3 to 5 days at 37°C under anaerobic conditions. Species identification was achieved using a Biotyper matrix-assisted laser desorption ionization–time of flight (MALDI-TOF) mass spectrometer (Bruker, Bremen, Germany). The isolates were stored at −80°C until further use.

### Sequencing.

Cultured bacteria were immersed in 0.9% phosphate-buffered saline to a McFarland standard of 0.5. DNA was extracted using a MagNA Pure 96 instrument (Roche, Basel, Switzerland). Libraries were prepared as described previously, and sequencing was performed on a NextSeq 500 sequencing system with a 300-cycle midoutput kit (Illumina, San Diego, CA, USA) (38). A mean of 1.2 million paired-end, 150-nucleotide (nt) reads were generated. Bases with a quality score less than Q30 and adapter sequences were trimmed, and any reads shorter than 36 nt were removed using Trimmomatic v0.36 (40). The remaining reads were used as input for the SPAdes v3.7.1 assembler (41), resulting in a mean coverage of 91.7× (maximum, 995.7×; minimum, 13.8×) and a mean *N*_50_ contig length of 348 kb. The sequencing data are publicly available in the NCBI Sequence Read Archive (SRA) database (see “Data availability,” below).

### Genotyping and genome-wide association study.

For typing purposes, the multilocus sequence type (MLST), clonal complex (CC), and *C. acnes* type or subspecies were determined using PubMLST (see Data set S1 in the supplemental material) (42). A phylogenetic tree was constructed using RAxML-NG (43). A bootstrap of 1,000 replicates was performed (44). The initial tree for the heuristic search was randomly generated. A discrete gamma distribution was used to model evolutionary rate differences among sites with four categories. The analysis was based on a sequence alignment with a total length of 26,933 bases. The association of genes with infection or commensalism was analyzed with an open reading frame (ORF)-based genome-wide association study (GWAS), as described previously (38). Briefly, genomes were annotated using PROKKA v1.14.5 (45), a pangenome was constructed from all isolates included in this study, and then a gene presence/absence matrix was built using Roary v3.13.0 (46) with default settings (sequence identity, 95%) for protein alignment. The association of each gene in the pangenome with infection or commensalism was tested using Scoary v1.6.16 with default settings (47). Scoary allows scoring of each candidate gene with respect to its association with the phenotype (infection/commensalism). There is an initial screen for associated loci. Positive hits from that first screen are then reanalyzed in a second round incorporating information on the phylogenetic structure of the input isolate collection. More specially, a phylogenetic tree was constructed, and its tips were annotated with trait and gene status at each step. By recursively traversing the tree, Scoary was used to compute the number of times the trait and gene coemerged for each gene from the initial screen for further pairwise comparisons (48). A *post hoc* permutation test ensured the validity of the results (47). A Benjamini-Hochberg adjustment for multiple testing was conducted (49). ORFs with a native *P* value of below 0.01 were annotated using GAMOLA2 (50).

### Data availability.

The sequencing data are publicly available in the NCBI Sequence Read Archive (SRA) database under the BioProject accession number PRJNA929346 and SRA accession numbers SRR23269205 to SRR23269393.
