# Supplementary material for: Genomics of Invasive Cutibacterium acnes Isolates from Deep-Seated Infections
Source: Microbiol Spectr. 2023 Mar 28;11(2):e04740-22. doi: 10.1128/spectrum.04740-22 (PMC10100948; doi:10.1128/spectrum.04740-22)
Supplement: Supplemental file 4 — Supplemental material. Download spectrum.04740-22-s0001.pdf, PDF file, 0.3 MB [file spectrum.04740-22-s0001.pdf]

Both et al., : Invasive *Cutibacterium acnes* populations supporting figures

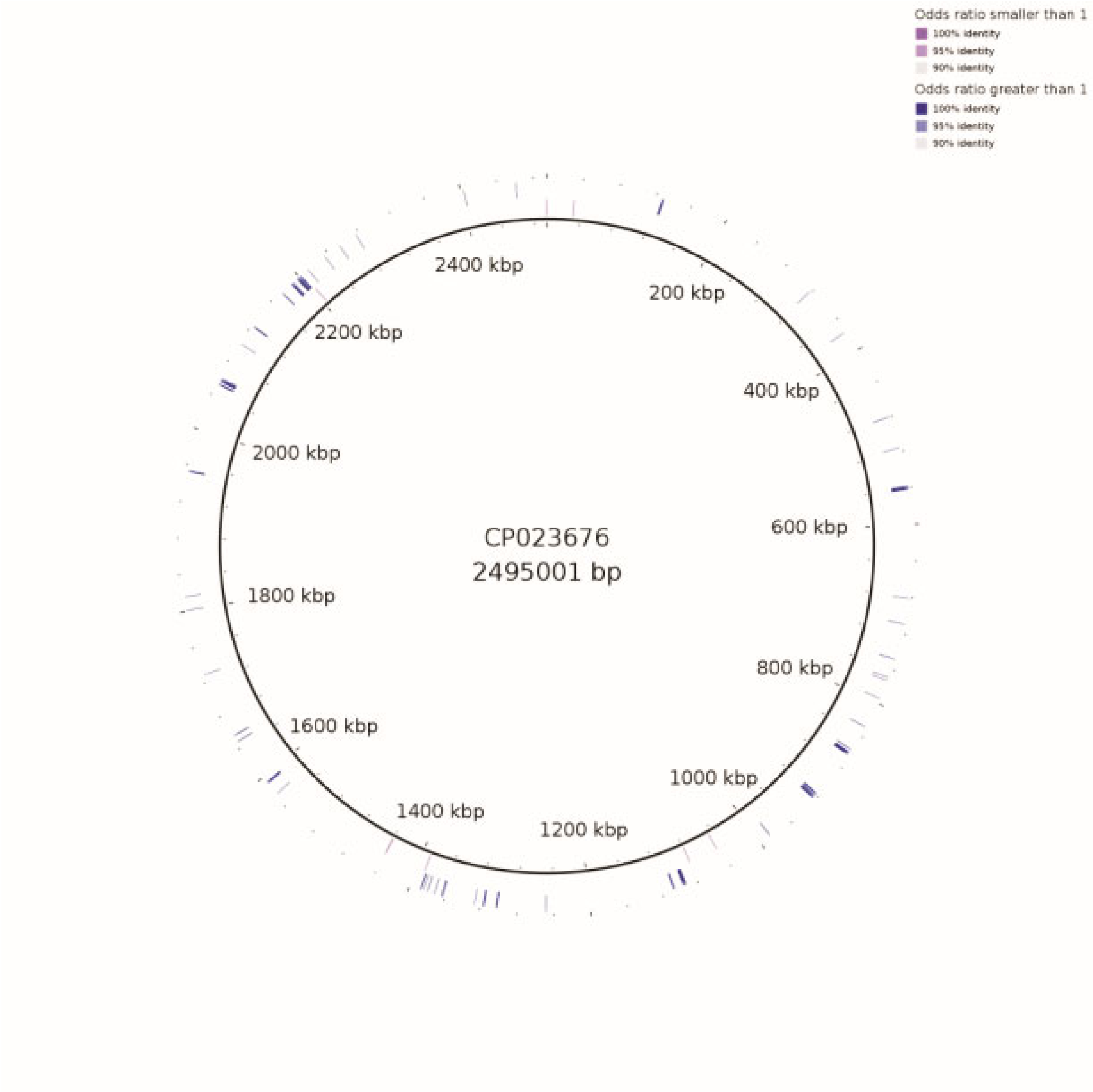

Figure S1: Alignment of significantly associated ORFs (GWAS) to genome of *C. acnes* ATCC 6919, CP023676.1, phylotype $IA_{1.}$

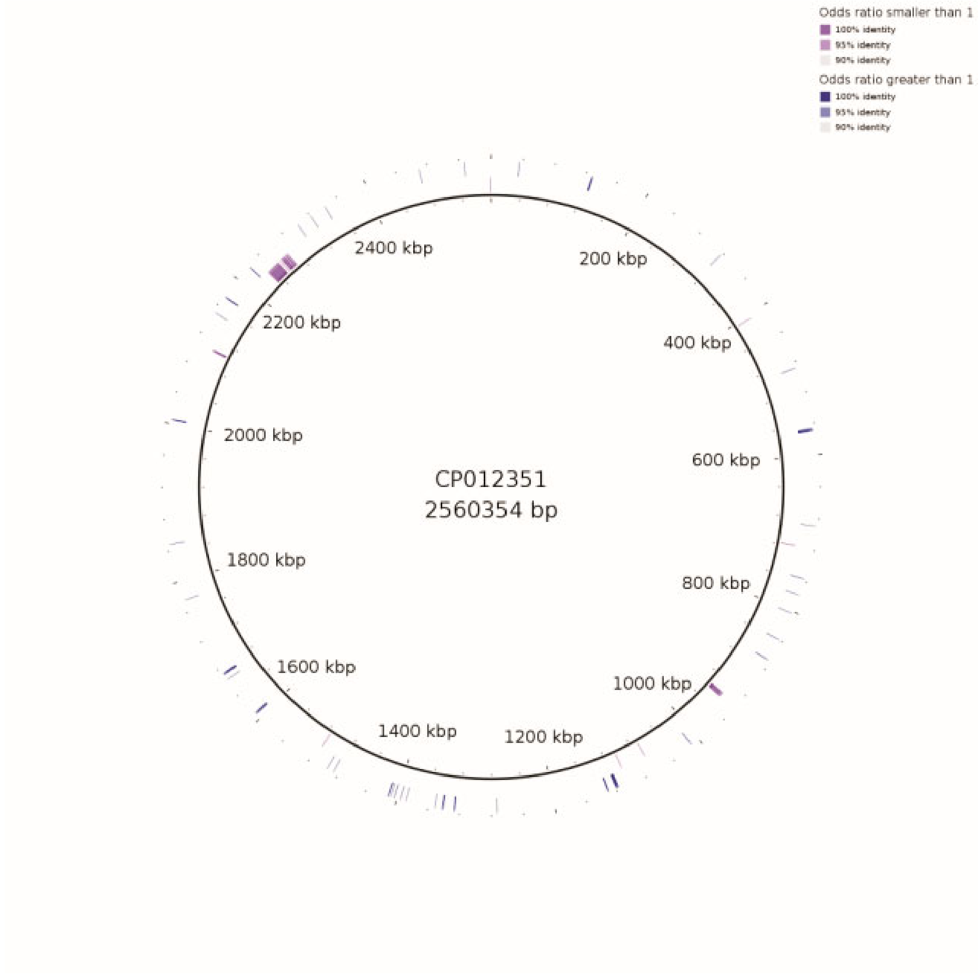

Figure S2: Alignment of significantly associated ORFs (GWAS) to genome of *C. acnes* PA15_2_L1, CP012351.1, phylotype IB.

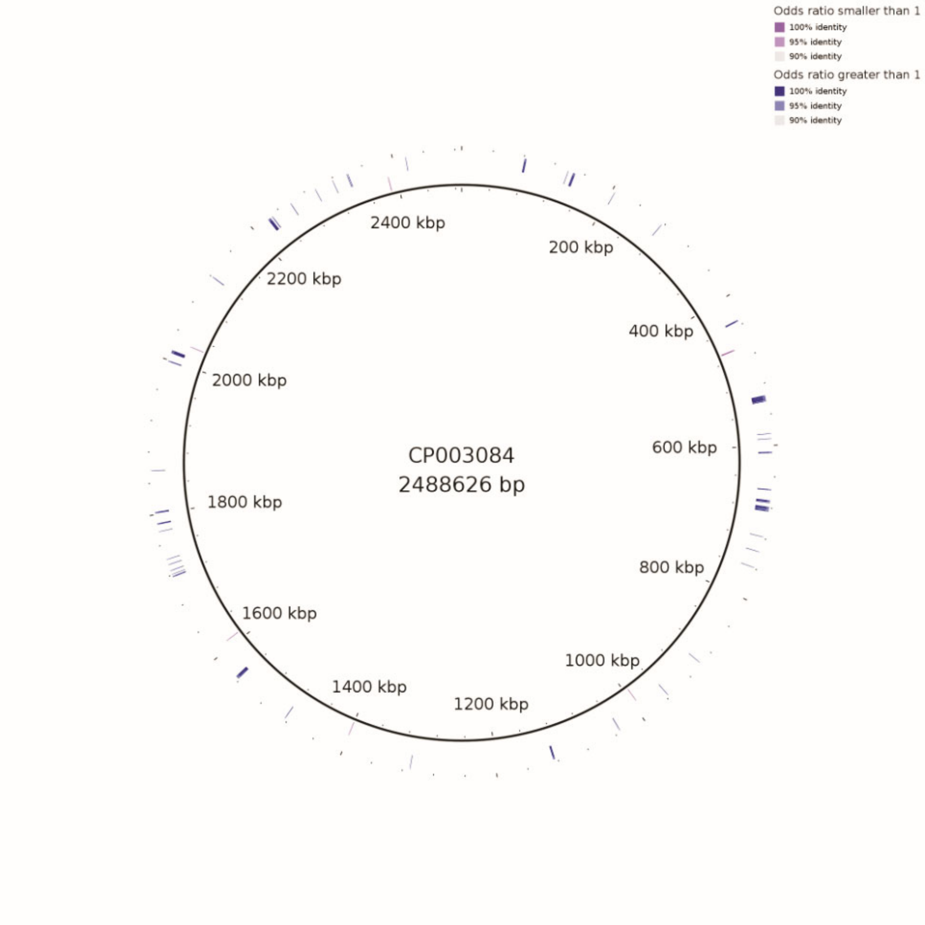

Figure S3: Alignment of significantly associated ORFs (GWAS) to genome of *C. acnes* ATCC 11828, CP003084.1, phylotype II (*C. acnes ssp. defendens*).
